# Method of preparing an equimolar DNA mixture for one-step DNA assembly of over 50 fragments

**DOI:** 10.1038/srep10655

**Published:** 2015-05-20

**Authors:** Kenji Tsuge, Yukari Sato, Yuka Kobayashi, Maiko Gondo, Masako Hasebe, Takashi Togashi, Masaru Tomita, Mitsuhiro Itaya

**Affiliations:** 1 Institute for Advanced Biosciences, Keio University, 403-1 Nipponkoku, Daihoji, Tsuruoka, Yamagata 997-0017, Japan

## Abstract

In the era of synthetic biology, techniques for rapidly constructing a designer long DNA from short DNA fragments are desired. To realize this, we attempted to establish a method for one-step DNA assembly of unprecedentedly large numbers of fragments. The basic technology is the Ordered Gene Assembly in *Bacillus subtilis* (OGAB) method, which uses the plasmid transformation system of *B. subtilis*. Since this method doesn’t require circular ligation products but needs tandem repeat ligation products, the degree of deviation in the molar concentration of the material DNAs is the only determinant that affects the efficiency of DNA assembly. The strict standardization of the size of plasmids that clone the DNA block and the measurement of the block in the state of intact plasmid improve the reliability of this step, with the coefficient of variation of the molar concentrations becoming 7%. By coupling this method with the OGAB method, one-step assembly of more than 50 DNA fragments becomes feasible.

The emergence of several examples of the reconstruction of genome-sized DNA^1^,^2^,^3^ has been stimulating the demand for genomic DNA with a *de novo*−designed sequence. The creation of a novel genomic DNA, however, still depends on chemical synthesis, which generates unsatisfactory DNA in terms of both size and the fidelity of construction^4^. As a result, these DNAs would require multiple assembly and sequencing steps to obtain a genome-sized DNA. To reduce the number of iterations required, a method for assembling large numbers of DNA fragments simultaneously is desired.

The Ordered Gene Assembly in *Bacillus subtilis* method (OGAB), developed in 2003, can efficiently assemble a dozen DNA fragments at once^5^,^6^,^7^,^8^,^9^,^10^. The method incorporates a *B. subtilis* plasmid transformation system that includes several unique processes: a double-strand break of DNA on the cell surface, incorporation of either single-strand DNA from the cutting site, and cyclization of the DNA inside the cell^5^,^8^,^11^,^12^ (Fig. 1). Like other assembly methods, OGAB assembly connects DNA blocks according to the sequence identities of the ends of the two relevant DNA blocks. Except for a yeast system that harnesses multiple DNA fragments *in vivo*^13^,^14^, recent popular DNA assembly methods using *Escherichia coli* as a host, such as SLIC^15^, Golden Gate^16^,^17^, CPEC^18^, Gibson^19^, and SLiCE^20^, require *in vitro* connection (or recombination) products having a circular form. The mechanism underlying the formation of the circular product with multiple components is complicated, because two different types of connections, intramolecular and intermolecular, are both required. Indeed, the efficiency of intramolecular ligation is affected by a variety of factors, such as DNA fragment length^21^ and the concentrations of polyethylene glycol and cation^22^,^23^. However, it can be said that efficiency decreases as the assembly scale increases, because the timing of intramolecular ligation for a ligating product is limited to when the product has the same number of DNA components with the assembly scale or with just multiples of the assembly scale, while intermolecular ligation proceeds simultaneously irrespective of the preferable timing of intramolecular ligation. For example, in the case of 6-fragment assembly, the timing of cyclization for a ligating product might appear 1/6, while in the case of 51-fragment assembly the timing might appear to be only 1/51 over the ligation duration. This might explain why cyclization with multiple components is inefficient. In contrast, OGAB requires an *in vitro* ligation product possessing multiple assembly units in one molecule in the manner of a tandem repeat. This is achieved only by intermolecular ligation and is free from the timing problem (Fig. 1 and Supplemental Fig. S1).

The redundancy (*r*) of the tandem repeats of an *in vitro* ligation product is the most critical determinant of assembly accuracy and efficiency in the OGAB method. The minimum value required for *r* is more than 1, but higher *r* is preferable (Supplemental Fig. S1). To obtain a ligation product with higher *r*, the material DNA fragments, called OGAB blocks, should be close to equimolar concentrations, although in practical terms it is impossible to achieve complete equimolarity (Fig. 1B,C). In the original OGAB method, however, it would be laborious to adjust even a dozen OGAB blocks to a molar concentration as the coefficient of variation of molar concentration (CV_mol_) ≈ 20%, due to the difficulty of adjusting the molar concentration of OGAB blocks with size variations (Supplemental Fig. S2). To reduce the CV_mol_ value toward a larger-scale assembly, we must strictly standardize the size of all OGAB blocks to an almost uniform length irrespective of genetic function, and then clone the blocks into the same vector (Fig. 2). From this, two advantageous features emerged. One is that this standardization makes it feasible to adjust the molar concentration of an OGAB block in the state of an intact cloning plasmid (hereafter we refer to this as OGAB block plasmid). Thanks to the precise stoichiometric magnification of the weight concentrations of the OGAB blocks by the vector portion, the same weight concentration between OGAB block plasmids can be regarded as the same molar concentration between relevant OGAB blocks. It is also expected that even if there is a certain degree of coefficient of variation of the sizes (CV_size_) of the OGAB blocks, the deviation would be buffered by using a CV_size_ of the full size of the plasmid. The other advantage is that OGAB blocks after restriction digestion can be separated from the vector portions in a single electrophoresis by treating the integrated equimolar mixture as one solution.

To demonstrate the merits that come from the strict standardization of OGAB block size, we performed two types of DNA assemblies: reconstruction of the lambda phage genome and synthesis of a *de novo*−designed operon. Each assembly is comprised of more than 50 OGAB blocks. Additionally, the theoretical aspects and the feasibility of larger-scale assembly using this improved method are discussed.

## Results

### Design outline of the OGAB blocks for lambda phage genome reconstruction

To demonstrate the performance of this improved method, we applied it to the reconstruction of 48.5 kb of a complete lambda phage genome (Fig. 3A). We designed the OGAB blocks by taking the following points into account. First, the blocks should be different in size from the cloning vector, because the they must be completely separated from the vector by a single electrophoresis. The next point concerns the selection of restriction enzymes to produce OGAB blocks with short protrusions at both ends that are responsible for the ligation order and orientation by T4 DNA ligase. Type IIS restriction endonuclease, which digests DNA outside of the recognition sequence, was designed to prepare OGAB blocks without leaving any trace of the restriction enzyme recognition sequence (Supplemental Fig. S3). Especially, a series of Type IIS restriction endonucleases that generate a protrusion with an arbitrary 4-nucleotide sequence at the 5’ end was considered useful for the excision of OGAB blocks. The size and number of possible OGAB blocks were simulated by varying both the combination of enzymes and the lengths of potential OGAB blocks to achieve a certain solution in which every OGAB block was assigned a restriction enzyme that did not cut inside of the relevant OGAB block. As a result, 48.5 kb of the lambda genome DNA was determined to be dissected into 50 OGAB blocks averaging 970 bp in length, which were prepared by any of three restriction enzymes: AarI, BbsI, and BsmBI (Fig. 3A).

### Precise design of 50 OGAB blocks for seamless assembly

The protrusions should be unique at each junction in one assembly. We chose 60 pairs of 4-base 5’ protrusions in advance as candidates according to the nucleotide compositions and sequence. These 60 pairs are comprised from 44 protrusion pairs that contain two A or T and two C or G nucleotides, but not palindromes, and 16 protrusion pairs that contain one A or T and three C or G nucleotides where the three C or G are not thrice in a row (Supplemental Materials and Methods). To realize seamless assembly, the 4-base protrusion candidate was searched from a sequence around the ideal dissection border (970 bp pitches) in the objective sequence (Supplemental Materials and Methods, Supplemental Table S1). We assigned the specific protrusion by computer simulation to minimize the size deviation of 50 OGAB blocks as CV_size_ = 0.65% (970.4 ± 6.3 bp, average ± standard deviation) (Supplemental Materials and Methods, Supplemental Table S2). The selected restriction enzyme site was added at each end of the OGAB block in a convergent manner with the block in order to generate the specific protrusion sequences for the PCR amplification (Supplemental Fig. S3).

### Purification of the OGAB block plasmids

The OGAB blocks thus designed were cloned into a conventional *E. coli* cloning plasmid, pMD19 (2,692 bp; Takara), and confirmed by sequencing. At this time point, the CV_size_ of the 50 OGAB block plasmids was only 0.18% (3691.4 ± 6.5 bp). Crude plasmid extracted from *E. coli* culture using a kit (QIAprep Miniprep Kit; Qiagen) would have at most several dozen percent of contaminated genome DNA (Supplemental Fig. S4). To eliminate this, the plasmid was further purified using Plasmid-Safe DNase (Epicenter), an ATP-dependent exonuclease (Supplemental Fig. S4). The weight concentrations of the obtained pure OGAB block plasmids were measured by a microvolume spectrophotometer known to have 2% measurement error under normal conditions (NanoDrop 2000; Thermo). They were then adjusted to 100 ng/μl by dilution with TE [10 mM Tris·HCl (pH 8.0), 1 mM EDTA], and the actual concentration was ascertained by repeated measurement with the microvolume spectrophotometer.

### Preparation of equimolar OGAB blocks

**Figure 2:**
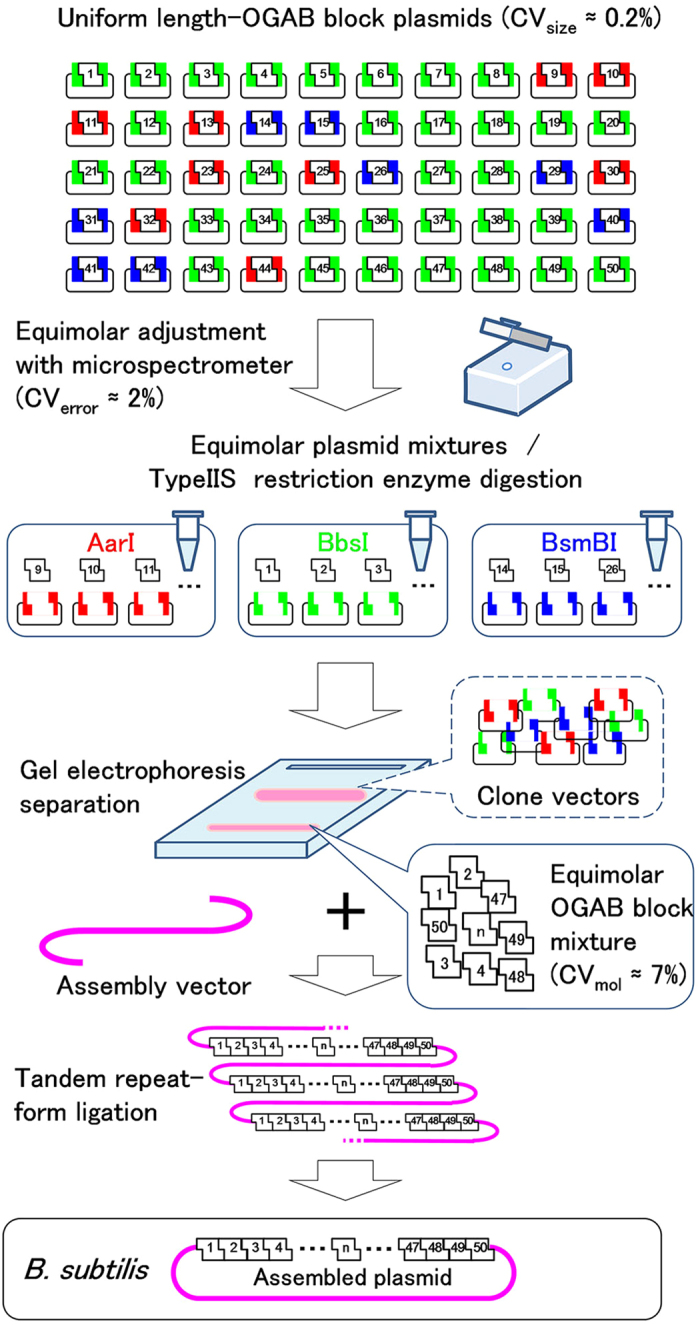
Overall features of objective DNA construction by OGAB methods from planetary (50 or more) OGAB blocks. The OGAB blocks, which are designed to have almost uniform lengths (CV_size_ ≈ 0.2%) and unique protrusions at both ends, were cloned into plasmid vectors interspaced with appropriate Type IIS restriction enzyme sites in a convergent manner (shown in red, blue, or green). After sequence confirmation, the molar concentrations of the blocks were adjusted in the state of OGAB block plasmid by a microvolume spectrophotometer with minimized error (CV_error_ ≈ 2%). The equimolar mixers of OGAB block plasmids, which were sorted according to the restriction enzyme used, were digested with the respective enzymes. The enzymes were then inactivated, and the mixtures were integrated into one tube and size-selected by electrophoresis. The obtained OGAB blocks retained an equimolar relation such as CV_mol_ ≈ 7%. They were added to an equimolar amount of the assembly vector, ligated into tandem-repeat linear form DNA, and then used to transform *B. subtilis*. Finally, objective DNA was obtained as plasmid DNA.

**Figure 3:**
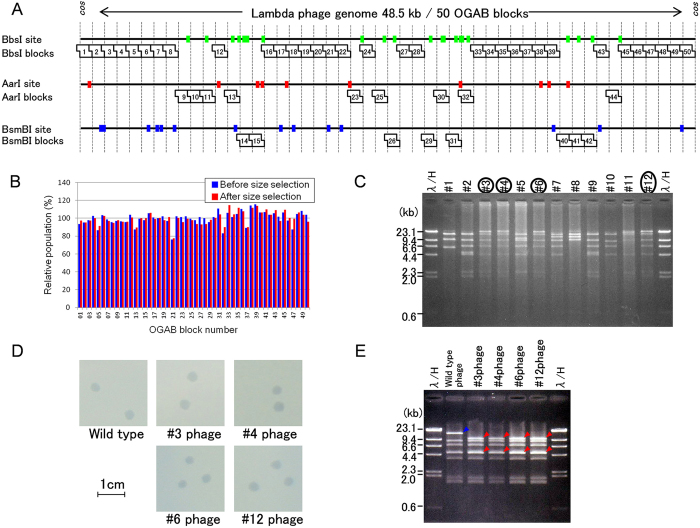
Lambda phage genome construction. (**A**) Design of the OGAB blocks. A total of 48.5 kb in length of lambda phage genome was divided into 50 fragments, each of which was cloned into cloning vector pMD19. Three restriction enzyme sites, BbsI (green), AarI (red), and BsmBI (blue), at least one of which did not appear in each OGAB block, were used. (**B**) The 50 OGAB block mixtures before (blue) and after (red) size selection by electrophoresis were compared relative to the population by quantitative PCR. The apparent CV_mol_ values for the OGAB blocks before and after size selection were 7.4% and 7.0%; however, due to the 3.6% measurement error of the PCR machine, the error-corrected CV_mol_ values were 7.0% and 6.6%, respectively. The population profile after size selection was almost the same as that before size selection. We performed two additional OGAB block preparations using independently measured and mixed OGAB block plasmids and determined CV_mol_(%), resulting in a similar value to that of the initial experiment (CV_mol_(%)(error-corrected) = 6.8% and 7.3%). All raw data are indicated in Supplemental Table S8. (**C**) Restriction digestion pattern of plasmids from 12 randomly selected transformants. HindIII and SfiI were used for double digestion. In the case of four clones (numbers circled), except for the 15 kb of the assembly vector pGET118-AarI, all of the bands were the same as the commercial size marker λ/HindIII. (**D**) Plaque formation assay of correctly assembled plasmid. The four plasmids were digested with lambda terminase, packed into lambda phage extract, and used to infect *E. coli*. There were no differences in features between the assembled plasmid-born plaque and the authentic lambda phage DNA-born plaque. (**E**) Confirmation of the plaque as designed. Due to an intended synonymous codon mutation in OGAB block 10, a restriction enzyme AvaI site appeared at the largest fragment (14,678 bp) of the wild type, generating two fragments (9,885 and 4,793 bp). All of the clones had restriction patterns at most large AvaI fragments distinct from those of the wild type, indicating that these phages originated from assembled DNAs.

Five hundred nanograms of each plasmid was transferred into one of three tubes that were marked AarI, BbsI, or BsmBI, according to plasmid design. The restriction enzyme AarI, BbsI, or BsmBI was added to the corresponding tube and incubated at 37 °C (or 55 °C for BsmBI) for 2 h (Fig. 2). After incubation, the same volume of phenol-chloroform-isoamyl alcohol (25:24:1, pH7.9) was added to each reaction and vortexed to inactivate the enzyme. The water-phenol mixtures of the three tubes were integrated into one tube, followed by phenol extraction and subsequent ethanol precipitation. At this point, we confirmed that the mixture was really sufficiently equimolar in concentration, based on a CV_mol_ value of 7.0% or less (as a measurement-error-corrected value) by quantitative PCR (Fig. 3B). The resultant DNA was then size-selected by electrophoresis using low-gelling-temperature agarose gel (Fig. 2). The obtained OGAB block mixture was confirmed to be perfectly retained as a 6.6% CV_mol_ (Fig. 3B).

### Assembly of 51 OGAB blocks

A solution containing 50 OGAB blocks at an equimolar concentration was added to an equimolar amount of the *B. subtilis* vector plasmid pGETS118-AarI (Supplemental Fig. S5) to obtain a 3 fmol/μl equimolar mixture. Ten microliters of this mixture was added to 11 μl of 2 × ligation buffer and 1 μl of T4 DNA ligase (4 Weiss units/μl; Takara), and then ligated at 37 °C for 4 h (Supplemental Fig. S6). The resulting ligation product was used to transform competent cells of *B. subtilis* BUSY9797^9^. Among the 230 tetracycline-resistant transformants that appeared, 12 clones were randomly selected, of which 4 showed the correct restriction pattern (Fig. 3C). We confirmed that the nucleotide sequences of all four of these clones were completely identical to the designed one. Furthermore, to confirm the biological activity of these four clones, their plaque formation ability was tested and confirmed to be normal (Fig. 3D). We also confirmed the existence of intended mutation in the phage genome DNA (Fig. 3E). Thus, we demonstrated that this improved method could feasibly be used to assemble more than 50 OGAB blocks.

### *De novo* construction of artificial mevalonate operon from 55 OGAB blocks

**Figure 4:**
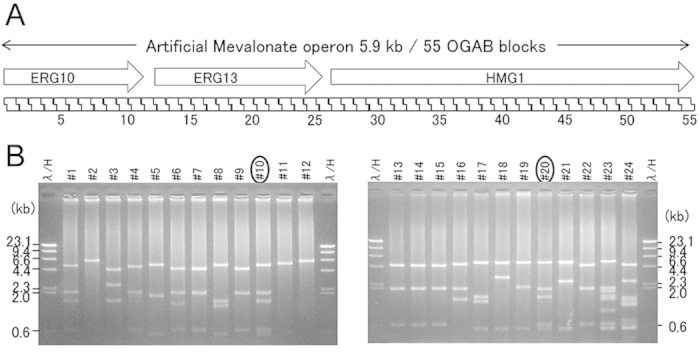
(**A**) *De novo* design of an artificial mevalonate operon. Three genes, ERG10, ERG13, and HMG1, from *Saccharomyces cerevisiae* whose codon usage was converted for *E. coli* expression, were connected to form an artificial operon. This sequence was divided into 55 OGAB blocks. (**B**) PvuII digestion patterns of plasmids from 24 randomly selected transformants. Two clones (numbers circled) were further confirmed to be correct by the restriction enzyme patterns of 13 kinds of enzymes and by sequencing.

To further confirm the feasibility of this method, we attempted to construct a completely artificial sequence from short synthetic oligonucleotides. An artificial mevalonate operon with a *de novo*−designed sequence of 5,951 bp, which originally was yeast genes but was optimised for *E. coli* codon usage, was constructed (Fig. 4A). Fifty-five OGAB blocks with a CV_size_ value = 4.2% (108 ± 4.5 bp) were designed (Supplemental Table S3 and S4). OGAB blocks constructed from synthetic oligo DNAs by the method of Rossi^24^ using the AarI site for protrusion generation were cloned into pMD19 and sequenced. Actually, in the state of OGAB block plasmids the difference was buffered to only CV_size_ = 0.16% (2828.2 ± 4.5 bp). The obtained equimolar amount of 55 OGAB blocks and assembly vector pGETS151-AarI were ligated (Supplemental Fig. S7) and used for *B. subtilis* transformation. Among the 154 colonies formed on the selection plate, plasmids of 24 randomly selected colonies were checked. Of these, two had the expected restriction digestion pattern (Fig. 4B), and the sequence integrity of each was confirmed by sequencing.

## Discussion

**Figure 5:**
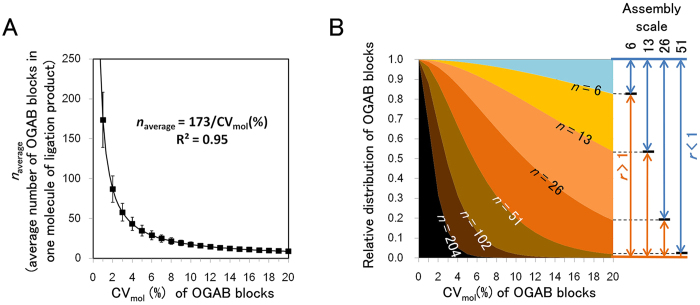
(**A**) Relationship between CV_mol_ and the average number of OGAB blocks in one ligation product (*n*_average_). The *n*_average_ value for each CV_mol_(%) was calculated from all relevant simulation results irrespective of the assembly scale and plotted as a square symbol with standard distribution (bars). The fitting curve in the graph represents all of the results with the R^2^ (square of the correlation coefficient) = 0.95. (**B**) Effect of CV_mol_ of the OGAB blocks on the population distribution of the *in vitro* ligation products in terms of the content rate of OGAB blocks. This figure was drawn using Equation 6. Ligation products with higher *n* values are located closer to the bottom of the figure. The lines indicate the contours for the indicated *n* values. For example, *n* = 6, 13, 26, and 51 indicate borders of *r* = 1 for the 6-, 13-, 26-, and 51-fragment assemblies, respectively.

As shown above, we successfully demonstrated gene assembly from more than 50 DNA fragments in one step. The key point must be the redundancy (*r*) of the tandem repeats of an *in vitro* ligation product, but there is no information about the allowable fluctuations in concentration. To assess this, computer simulations of the *in vitro* ligation were performed using hypothetical fragments containing an average of 640 molecules for identical OGAB blocks with defined CV_mol_. The simulation was performed under the condition that only intermolecular ligation was permitted; intramolecular ligation was prohibited since our adopted ligation condition doesn’t form circular DNA^5^,^22^,^23^. The ligation simulation ran until all canonical ligation pairs were exhausted (Supplemental Fig. S8). For each assembly scale (6, 13, 26, and 51) and for each CV_mol_ (from 0 to 20%, 1% interval), 20 simulations were performed using an independently prepared randomised initial value, resulting in a total of 1,680 simulation plots (Supplemental Fig. S8). Figure 5A shows the effect of the degree of CV_mol_ of OGAB blocks on *n* (the number of OGAB blocks in one molecule of ligation product), which summarises the simulation results. A fitting curve for *n*_average_ (average *n* value) revealed that this parameter can be described simply according to the following equation, regardless of assembly scale:


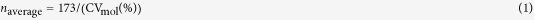


This equation means that when CV_mol_ of prepared OGAB blocks is 1%, we can expect ligation products accommodating 173 OGAB blocks on average, whereas when CV_mol_ is 20%, the expected block number drops to only 8.6. Because this equation doesn’t give any information about distribution, we then analysed the population distribution of *n* for each ligation simulation condition. The outline of distributions, at a glance, was exponential distribution such as a monotonic decrease as a function of *n*. However, ligation products were periodically absent where *n* equalled to just multiples of the fragment number of assembly. This is obviously different from a canonical exponential profile. Exponential distribution *f*(*n*) is expressed as a function of *n* as follows:


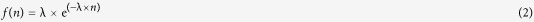


where λ is the rate parameter of exponential distribution. Since the logarithmic transformation of Equation 2 is as follows:


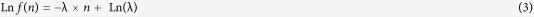


λ can be calculated from the slope of a linear approximation of plots on *n*-Ln*f*(*n*), with Ln*f*(*n*) corresponding to the logarithmic-transformed count of ligation products in Supplemental Fig. S8B. Hereafter, λ calculated this way is denoted as λ_slope_. The λ_slope_ values obtained from all of the simulation conditions were plotted on a CV_mol_-λ_slope_ coordinate (Supplemental Fig. S8C), revealing that λ_slope_ can be expressed as a function of CV_mol_ almost regardless of the difference in assembly scales, as follows:


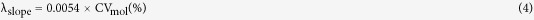


On the other hand, λ can be calculated in a different way using the average value of exponential distribution, which corresponds to *n*_average._ Hereafter, this λ is presented as λ_average_. According to Equation 1, λ_average_ can be converted as follows:


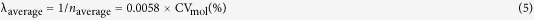


Since λ_slope_ closely resembles λ_average_, it can be said that this profile well follows an exponential distribution. Thus we heuristically regarded this ligation product population profile as exponential distribution, and performed further simulation using the following approximation by choosing λ_average_ as a rate parameter.


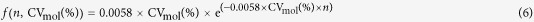


This equation simply indicates that the value of CV_mol_ is the only determinant for the distribution, regardless of the assembly scale (Fig. 5). Since the value of *r* is given as:


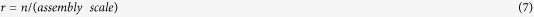


This equation means that as the assembly scale becomes larger, the precision of the adjustment of the OGAB blocks to an equimolar concentration would increase in an inversely proportional manner; in the case of a 6-fragment assembly, even though the fluctuation was as high as CV_mol_ = 20%, 82% of the OGAB blocks were incorporated into the ligation product with *r* > 1. However, if a 60-fragment assembly is projected, it might require CV_mol_ = 2% for the same ligation profile of a 6-fragment assembly, meaning a 10-fold greater precision would be needed.

**Figure 6:**
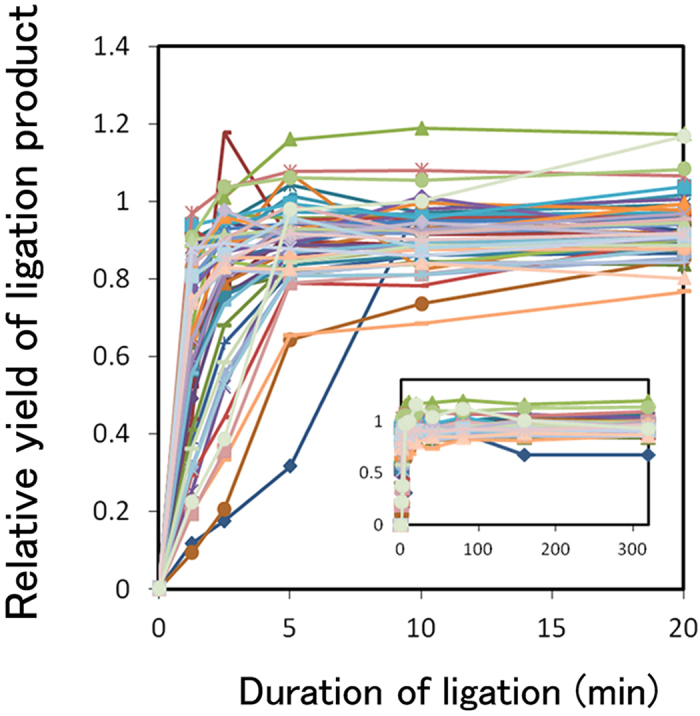
Time course of the ligation yield of all junctions in lambda genome reconstruction.To check the ligation yield, all 51 ligation junctions of the lambda phage reconstruction were monitored by quantitative PCR, and are plotted in different colours or using different symbols (not specified). Samples were collected at 0, 1.25. 2.5, 5, 10, 20, 40, 80, 160, and 320 min after the ligation started. Plots of longer ligation duration are superimposed on those of shorter ligation duration. Ligation was almost finished within 4 h and 20 min, which was our adopted condition for assembly, and this duration was considered sufficient to complete the ligation. Raw data are indicated in Supplemental Table S9.

**Figure 7:**
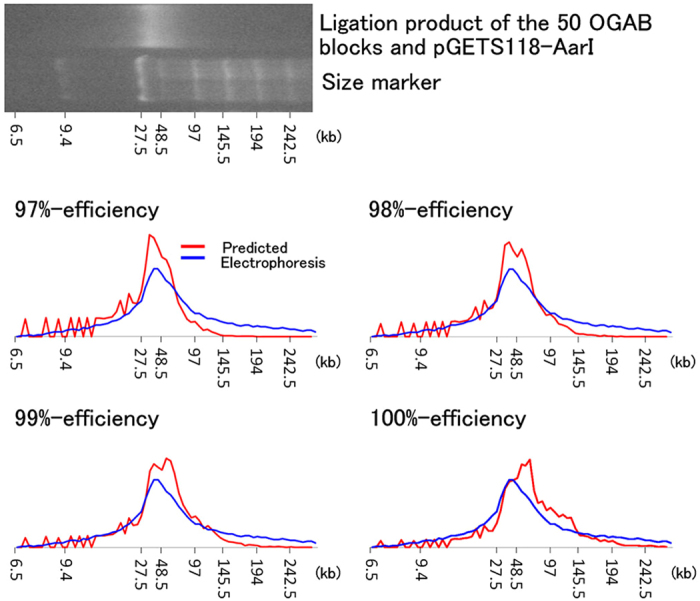
Assessment of the simulation. (**A**) Pulsed-field gel electrophoresis separation of the ligation products of 51 OGAB blocks for lambda phage reconstruction, where CV = 6.6% in molar concentration. Electrophoresis conditions: strength of electric field, 4.5 V/cm; pulse cycle, 30 s; running time, 11 h. (**B**) Comparison of the simulated distribution (red line) with the density distribution by electrophoresis (blue line). The results of four simulations that were performed until the canonical ligation pairs were exhausted (100% efficiency) or stopped at a point when 1%, 2%, or 3% of canonical ligation pairs remained unligated (99%, 98%, or 97% efficiency, respectively) are indicated. For each condition, 100 initial value sets, which included size information in bp for each OGAB block, were independently prepared. The obtained profiles were converted to a horizontal scale for comparison with the results of electrophoresis. The vertical direction indicates the relative intensities of fluorescence of ethidium bromide-stained DNA for electrophoresis. Both profiles are normalised in terms of area.

Of course, the results of computer simulations can only be considered useful for the quality control of OGAB blocks if they functions well in actual experiments. To assess this, a regression analysis for lambda genome reconstitution was performed. First, we analysed the inconsistency of molar concentration of OGAB blocks between the initial values calculated by optical absorption of 260 mm and the values measured by quantitative PCR (Fig. 3B). Since certain OGAB block plasmids showing lower DNA concentration by quantitative PCR in Fig. 3B would have higher optical adsorption ratio of 260 nm/230 nm at OGAB block plasmids level comparing with the rest of plasmids, it was probable that difference in impurity concentration among the OGAB block plasmids leads higher CV_mol_ value. We think that it isn’t easy to go lower CV_mol_ value than 7% in the current technology, because we couldn’t improve this situation even though performed three trials (Fig. 8B and Supplemental Table S4). Next, to identify the problems that led to the incorrect assembly of plasmids, all eight of the incorrect plasmids were sequenced, and the problems were attributed to misligation of OGAB blocks (Supplemental Fig. S9). However, the occurrence ratio was only 46 junctions per misligation junction, or nearly as low as one misligation per construct. On the basis of this result, we considered that the misligation problem did not seriously affect Equation 6. Next we investigated the ligation yield by kinetic analysis for all 51 junctions, and confirmed that all ligation reactions were completed under the conditions used (37 °C for 4 h) (Fig. 6). Finally, the actual length distribution of the *in vitro* ligation products that were used with OGAB blocks with CV_mol_ = 6.6% was compared with a distribution profile derived from a computer simulation using hypothetical OGAB blocks with the same CV_mol_ value (Fig. 7). The two profiles were almost identical in terms of the shapes of the simulation results at 98–100% ligation efficiency; for example, the maximum density of DNA appeared at around the 40 kb region. Thus we confirmed that the simulation results well described the actual experiment and would be applicable to the quality control of OGAB blocks. Notably, these results indicate that we may assemble multiple DNA fragments, as many as 100 at once, if we can control the concentration fluctuation within 3.3%.

The assembly is completed within 5 days if all OGAB plasmids are ready to use (Supplemental Table S5). Using this method, OGAB blocks can be freely designed in terms of both size and number, and the resulting blocks might be constructed using a synthetic DNA. The assembled DNA may be applicable to the production of genome-sized constructs using the *B. subtilis* genome vector platform^1^,^25^.

## Materials and methods

### Assembly vector

The assembly vector pGETS118-AarI-pBR is a shuttle vector between *B. subtilis* and *E. coli* constructed from pGETS118^26^ in several steps, as indicated in Supplemental Fig. S5. Two AarI sites, whose protrusion sequences are 5’-ATTA-3’/5’-TAAT-3’ and 5’-AAAA-3’/5’-TTTT-3’, were used for the assembly. Another assembly vector, pGETS151-pBR, was constructed by truncating plasmid origin of *B. subtilis* of pGETS118-AarI-pBR as indicated in Supplemental Materials and Methods. The obtained linearised plasmid DNAs then were named pGET118-AarI and pGETS151-AarI, respectively.

### Preparation of OGAB block plasmids

The OGAB blocks were amplified by PCR using the primers listed in Supplemental Table S6. PCR was performed using KOD DNA polymerase (Toyobo). An A-protrusion at the 3’ end was added to the obtained PCR fragment using A-attachment Mix (Toyobo) according to the instruction manual. The obtained DNA was ligated into pMD19 (simple) (Takara) using Mighty Mix ligation mixture (Takara), and then was used to transform each of the *E. coli* strains TOP10, JM109, and DH5α. Transformants of these plasmids were cultivated with LB medium supplemented with 100 μg/ml of carbenicillin (Wako, Japan). OGAB plasmid was purified from 1 ml of the culture using QIAprep Miniprep Kit (Qiagen) according to the instruction manual. The obtained crude plasmid solution was further purified enzymatically using Plasmid Safe DNase (Epicenter) as follows. Crude plasmid (5 μg/50 μl) was added to 6 μl 10 × buffer for Plasmid Safe DNase, 2.4 μl of 25 mM ATP, and 2 μl Plasmid Safe DNase. This reaction mixture was incubated at 37 °C for 1 h, then incubated at 70 °C for 30 min to inactivate the enzymes. The resulting solution was cleaned up by using a conventional column-based cleanup kit and then eluted in a small volume of TE (pH 8.0) (<25 μl).

### Preparation of equimolar mixture

A microvolume spectrophotometer determined the weight concentration of the purified OGAB block plasmid. Typically, the concentration ranged between 100 and 250 μg/μl. The purified OGAB block plasmid was then diluted to give 100 μg/μl solution with TE, after which the microvolume spectrophotometer confirmed that the concentration was very close to 100 μg/μl. The concentrations obtained here were used to calculate the precise volumes for just 500 ng to an accuracy of three significant digits.

### Restriction enzyme digestion

Restriction endonuclease AarI was obtained from Thermo. Restriction endonucleases BbsI and BsmBI were purchased from NEB. Restriction digestion of the plasmid mixture was performed while preserving the equimolar relation as follows. One volume of plasmid mixture was added to two volumes of sterilised water, 1/3 volume of 10 × buffer for the restriction enzyme, and 1/3 volume of the restriction enzyme. In AarI digestion, a manufacturer-supplied 50 × oligonucleotide solution was added for the preparation of OGAB blocks for the lambda phage, but it was not added for the preparation of blocks for the artificial mevalonate operon, in order to prevent contamination of the oligonucleotide in the OGAB blocks due to size similarity. The incubation temperature of BsmBI was 55 °C, while that of the other enzymes was 37 °C. After 2 h digestion at the appropriate temperature, 5% of the reaction mixture was sampled, and then 5 μl of that was checked for digestion integrity by electrophoresis.

### Inactivation of restriction enzymes

The enzymes in the mixtures were inactivated by adding the same volume of phenol-chloroform-isoamyl alcohol = 25:24:1 (Nakalai Tesque) and mixing. The resulting emulsions were integrated into one tube, followed by the usual phenol-chloroform-isoamyl alcohol extraction and subsequent ethanol precipitation. The supernatant was transferred to a new tube and subjected to phenol-chloroform-isoamyl alcohol. The supernatant was then transferred to another new tube, and mixed with 500 μl of 1-butanol and centrifuged at 20,000 × g for 20 s. After the upper butanol phase was removed, fresh butanol was added and the solution was further mixed. This dehydration was repeated until the water phase fell below 450 μl. The DNA was precipitated by the addition of 2.5 volumes of ethanol after 50 μl of potassium acetate (300 mM, pH 4.8) was mixed with the water phase. The precipitated DNA was rinsed with 70% ethanol, dried, and dissolved in 20 μl of TE.

### Size selection of OGAB blocks by agarose gel electrophoresis

Two types of agarose gels were used. As a low-gelling-temperature agarose, 2-Hydroxyethyl agarose (Sigma) was used. The electrophoresis was performed as follows: gel concentration, 0.7%; buffer, 1 × TAE [40 mM Tris-acetate (pH 8.3) and 1 mM EDTA]; strength of electric field, 3 V/cm; running time, 3 h. After staining with ethidium bromide, gel blocks containing the OGAB blocks were excised from the main part by 365 nm UV illumination. The excised gel block (up to 500 mg) was filled up to 650 μl with 1 × TAE buffer and melted at 65 °C for 15 min. The gel solution was added to 500 μl of phenol-saturated TE buffer (Nakalai Tesque) and mixed by a vortex, followed by centrifugation at 20,000 × g for 5 min. The supernatant was transferred to a new tube and subjected to phenol extraction until the gel debris that would be positioned at the surface between the phenol and water had disappeared. The supernatant was transferred to another new tube, then mixed with 500 μl of 1-butanol and centrifuged at 20,000 × g for 20 s. After removal of the upper butanol phase, fresh butanol was added and mixed in. This dehydration was repeated until the water phase fell below 450 μl. The DNA was precipitated by the addition of 2.5 volumes of ethanol after 50 μl of potassium acetate (300 mM, pH 4.8) was mixed with the water phase. The precipitated DNA was rinsed with 70% ethanol, dried, and dissolved in 20 μl of TE.

For the size selection of OGAB blocks for the artificial mevalonate operon, general agarose gel (UltraPure agarose; Invitrogen) was used in place of the low-gelling-temperature agarose, because short DNA is not tolerant to 65 °C. The electrophoresis was performed as follows: gel, 2.5% agarose with 1 × TAE buffer; strength of electric field, 3 V/cm; running time, 3 h. After separation, the gel was dissected into two parts, the main part and the size marker part, and only the latter was stained with ethidium bromide. After staining, gel blocks containing the OGAB blocks were excised from the main part by referring to the size of the stained gel by 365 nm UV illumination. The OGAB blocks were purified using a MinElute Gel Extraction Kit (Qiagen) according to the instruction manual, except that DNA was eluted with 10 μl of TE (pH 8.0) instead of the buffer included in the kit.

### Ligation for tandem-repeat linear form

A 2 × ligation buffer for linear form DNA [132 mM Tris·HCl (pH 7.6), 13.2 mM MgCl_2_ , 20 mM dithiothreitol, 0.2 mM ATP, 500 mM NaCl, 20% (w/v) polyethylene glycol 6000 (Wako Pure Chemical)] was prepared and used. The NaCl concentration in this buffer was higher than that of the former buffer. Ten microliters of this mixture (3 fmol/μl each) was added to 11 μl of 2 × ligation buffer, and 1 μl of T4 DNA ligase (4 Weiss units/μl) was added. After reaction at 37 °C for 4 h, 8 μl of ligation products was subjected to electrophoresis to confirm the ligation.

### Transformation of *B. subtilis*

Competent *B. subtilis* BUSY9797^9^ cells were prepared using the two-step culture method developed by Anagnostopoulos and Spizizen^27^. An overnight culture of *B. subtilis* (50 μl) in LB was inoculated onto 925 μl medium TFI containing 25 μl of 2% casamino acid. The TFI medium contained 1.4% K_2_HPO_3_, 0.6% KH_2_PO_4_, 0.2% (NH_4_)_2_SO_4_, 0.1% trisodium citrate, 0.5% glucose, 0.02% MgSO_4_·7H_2_O, 0.05 mg/ml tryptophan, 0.05 mg/ml arginine, 0.05 mg/ml leucine, and 0.05 mg/ml threonine. After shaking with vigorous aeration for 3.75 h at 37 °C, 100 μl culture was transferred into 900 μl of TFII medium. The TFII medium contained 1.4% K_2_HPO_3_, 0.6% KH_2_PO_4_, 0.2% (NH_4_)_2_SO_4_, 0.1% trisodium citrate, 0.5% glucose, 0.02% MgSO_4_·7H_2_O, 0.01% casamino acid, 0.005 mg/ml tryptophan, 0.005 mg/ml arginine, 0.005 mg/ml leucine, and 0.005 mg/ml threonine. After 90 min of incubation at 37 °C, a 100 μl aliquot of the cell culture was used for transformation.

### Plasmid extraction from *B. subtilis*

Colonies on a plate were picked up by a toothpick and inoculated into 2 mL of 10 μg/ml tetracycline-containing LB medium. After the culture reached the late-log to stationary phase at 37 °C, the plasmid copy number was amplified by the addition of IPTG (Isopropyl-β-D-thiogalactopyranoside) to the culture at a final concentration of 1 mM, followed by cultivation for another 3 h. The alkaline–SDS method described by Bron^28^ was used as indicated in Supplemental Materials and Methods.

## Author Contributions

K.T., M.T. and M.I. designed research; K.T., Y.S., Y.K. and M.G. prepared DNA fragments for assembly; K.T., M.I., M.H. and T.T. prepared assembly vectors; K.T., Y.S. and Y.K. performed assembly; and K.T., M.T. and M.I. wrote the paper.

## Additional Information

**How to cite this article**: Tsuge, K. *et al.* Method of preparing an equimolar DNA mixture for one-step DNA assembly of over 50 fragments. *Sci. Rep.* **5**, 10655; doi: 10.1038/srep10655 (2015).

## Supplementary Material

Supplementary Information

Supplementary Tables

## Figures and Tables

**Figure 1:**
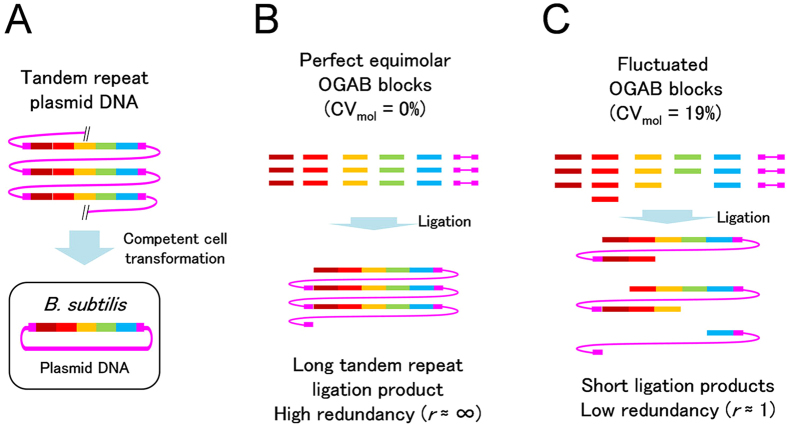
Conceptual explanation of the importance of equimolar preparation for OGAB assembly. (**A**) Plasmid transformation of *B. subtilis* competent cells. The donor plasmid DNA molecules should have a structure of tandem repeats in the plasmid unit, but the preparation of a circular plasmid is not required. DNA with a higher number of repeats is preferable (Supplemental Fig. S1). (**B**) Effect of the degree of coefficient of variation on the molar concentration (CV_mol_) of material DNAs on the number of repeat units in ligation products. If the material DNA is completely equimolar and has no fluctuation (CV_mol_ = 0%), all OGAB blocks may ligate in one molecule, resulting in DNA with a higher *r* value. (**C**) On the other hand, if there is a certain degree of fluctuation (e.g., CV_mol_ = 19%), the ligation products will result in fragments with a lower *r* value.
