# Supplementary material for: Method of preparing an equimolar DNA mixture for one-step DNA assembly of over 50 fragments
Source: Sci Rep. 2015 May 20;5:10655. doi: 10.1038/srep10655 (PMC4438487; doi:10.1038/srep10655)
Supplement: Supplementary Information [file srep10655-s1.pdf]

Supplemental File

Manuscript title: Method of preparing an equimolar DNA mixture for one-step DNA assembly of over 50 fragments

Authors: Kenji Tsuge, Yukari Sato, Yuka Kobayashi, Maiko Gondo, Masako Hasebe, Takashi Togashi, Masaru Tomita, and Mitsuhiro Itaya

Index of Supplemental Tables

(These tables appear in the Microsoft Excel file (.xls))

Supplemental Table S1. Statistics on the protrusion selection for 50-OGAB blocks for the lambda phage reconstruction.

Supplemental Table S2. The design of 50-OGAB blocks for lambda phage construction.

Supplemental Table S3. Primer sequence for the OGAB blocks for lambda phage reconstruction.

Supplemental Table S4. Relative molar concentrations of the OGAB blocks for lambda phage genome reconstruction.

Supplemental Table S5. Statistics of the protrusion selection for 55 OGAB blocks for the artificial nonmevalonate operon construction.

Supplemental Table S6. The design of 55 OGAB blocks for artificial mevalonate operon construction.

Supplemental Table S7. Primer sequences for the OGAB blocks for the artificial mevalonate operon construction.

Supplemental Table S8. Time course of the ligation yield of all junctions in the lambda genome reconstruction.

Supplemental Table S9. Typical time schedule of this method.

Supplemental Materials and Methods

Assembly vector construction. The assembly vector pGETS118-AarI-pBR is a shuttle vector between *B. subtilis* and *E. coli* constructed from pGETS118 (22) in several steps (Fig. 5S). Two AarI sites, whose protrusion sequences are 5'-ATTA-3'/ 5'-TAAT-3' and 5'-AAAA-3' / 5'-TTTT-3', were used for the assembly. Between the two sites, truncated pBR322 sequence, which had a replication origin and an ampicillin resistance gene, were inserted to amplify the copy number of the original pGETS118 at vector DNA preparation. The tetracycline resistance gene in pGETS118-AarI-pBR was modified from the original pGETS118 by introducing a point mutation to eliminate the naturally

occurring AarI site. Another assembly vector, pGETS151-pBR, was constructed from three PCR products that were obtained using pGETS118-AarI-pBR as template DNA by three primer sets: Part A (5'-TAGGGTCTCAAAGCGGCCGCAAGCTT-3' and 5'-TAGGGTCTCAGCGGCCAAGAAGGCC-3), Part B (5'-TAGGGTCTCACCGCCCTTCCCGGTCGATAT-3' and 5'-TAGGGTCTCATATTAGCTTAATTGTTATCCGCTCACAATTCC-3'), and Part C (5'-TAGGGTCTCAAATAACTGGAAAAAATTAGTGTCTCATGGTTCG-3' and 5'-TAGGGTCTCAGCTTAAGTGGTGGGTAGTTGACC-3'). The resulting fragments were digested with BsaI and then assembled by the OGAB method. This plasmid lacks functional regions for *E. coli* (between cat and *oriS*, and between *parA* and *parC*) (Fig. S5), but it functions the same as the original pGET118 at OGAB assembly. These plasmid vectors were digested with AarI for OGAB assembly under the following conditions: 10 µl of plasmid vector (corresponding to 5 µg), 29 µl of sterilised water, 5 µl of 10×Buffer_for_AarI, 1 µl of 50 × oligonucleotide, and 5 µl of AarI were mixed and then incubated at 37˚C for 2 h. The resulting DNA was separated by electrophoresis using low-gelling-temperature agarose. The assembly vector band was excised from the gel and extracted from agarose as indicated below. The DNA precipitation obtained was dissolved into 20 µl of TE, of which 1 µl was used to measure the concentration by the NanoDrop 2000 UV-vis spectrophotometer. The obtained linearised plasmid DNAs were then named pGET118-AarI and pGETS151-AarI.

Sequences of protrusions. The diversity of a 4-nucleotide sequence isis 4^4 (=256). There are 16 palindrome sequences. Because palindrome protrusions can ligate between identical fragments, they cannot be used for the OGAB method. Consequently, 240 nonpalindromic sequences include complementary pairs, thus 120 pairs exist. Any of the nonpalindromic sequences might be used for OGAB assembly, but we haven't tested all of them yet. In this study, except for the protrusion for the vector picked from group I, we chose 60 protrusions in advance according to a possible rule that potentially reflects ligation speed and fidelity, as follows: 44 protrusion pairs that contain two A or T and two C or G nucleotides but not are not palindromes (5'-AACC-3' / 5'-GGTT-3', 5'-AACG-3' / 5'-CGTT-3', 5'-AAGC-3' / 5'-GCTT-3', 5'-AAGG-3' / 5'-CCTT-3', 5'-ACAC-3' / 5'-GTGT-3', 5'-ACAG-3' / 5'-CTGT-3', 5'-ACCA-3' / 5'-TGGT-3', 5'-ACCT-3' / 5'-AGGT-3', 5'-ACGA-3' / 5'-TCGT-3', 5'-ACTC-3' / 5'-GAGT-3', 5'-ACTG-3' / 5'-CAGT-3', 5'-AGAC-3' / 5'-GTCT-3', 5'-AGAG-3' / 5'-CTCT-3', 5'-AGCA-3' / 5'-TGCT-3', 5'-AGGA-3' / 5'-TCCT-3', 5'-AGTC-3' / 5'-GACT-3', 5'-AGTG-3' / 5'-CACT-3', 5'-ATCC-3' / 5'-GGAT-3', 5'-ATCG-3' / 5'-CGAT-3', 5'-ATGC-3' / 5'-GCAT-3', 5'-ATGG-3' / 5'-CCAT-3', 5'-CAAC-3' / 5'-GTTG-3', 5'-CAAG-3' / 5'-CTTG-3', 5'-CACA-3' / 5'-TGTG-3', 5'-CAGA-3' / 5'-TCTG-3', 5'-CATC-3' / 5'-GATG-3', 5'-CCAA-3' / 5'-TTGG-3', 5'-CCTA-3' / 5'-TAGG-3', 5'-CGAA-3' / 5'-TTCG-3', 5'-CGTA-3' / 5'-TACG-3', 5'-CTAC-3' / 5'-GTAG-3', 5'-CTCA-3' / 5'-TGAG-3', 5'-CTGA-3' / 5'-TCAG-3', 5'-CTTC-3' / 5'-GAAG-3', 5'-GAAC-3' / 5'-GTTC-3', 5'-GACA-3' / 5'-TGTC-3', 5'-GAGA-3' / 5'-TCTC-3', 5'-GCAA-3' / 5'-TTGC-3', 5'-GCTA-3' / 5'-TAGC-3', 5'-GGAA-3' / 5'-TTCC-3', 5'-GGTA-3' / 5'-TACC-3', 5'-GTCA-3' / 5'-TGAC-3', 5'-GTGA-3' / 5'-TCAC-3', and 5'-TCCA-3' / 5'-TGGA-3'), and 16 protrusion pairs that contain one A or T and three C or G nucleotides where the three C or G aren't thrice in a

row (5’-CACC-3’ / 5’-GGTG-3’, 5’-CCAC-3’ / 5’-GTGG-3’, 5’-CTCC-3’ / 5’-GGAG-3’, 5’-CCTC-3’ / 5’-GAGG-3’, 5’-CACG-3’ / 5’-CGTG-3’, 5’-CCAG-3’ / 5’-CTGG-3’, 5’-CTCG-3’ / 5’-CGAG-3’, 5’-CCTG-3’ / 5’-CAGG-3’, 5’-CAGC-3’ / 5’-GCTG-3’, 5’-CGAC-3’ / 5’-GTCG-3’, 5’-CTGC-3’ / 5’-GCAG-3’, 5’-CGTC-3’ / 5’-GACG-3’, 5’-GAGC-3’ / 5’-GCTC-3’, 5’-GGAC-3’ / 5’-GTCC-3’, 5’-GTGC-3’ / 5’-GCAC-3’, and 5’-GGTC-3’ / 5’-GACC-3’).

Designing OGAB blocks for lambda phage genome reconstruction. The design of OGAB blocks would be affected by the distribution of naturally occurring Type IIS restriction enzyme sites. The Type IIS enzymes AarI, BbsI, BfuAI, BsmFI, and BtgZI were selected for candidate enzymes, since there is no cutting site in subcloning vector pMD19 (Takara Bio). In addition, the Type IIS enzyme BsmBI was also included among the candidates, since the three fragments of pMD19 generated by this enzyme are small enough and large enough against possible OGAB blocks (0.7 to 1.5 kb) at electrophoresis. The restriction sites of these enzymes appeared to multiply through lambda DNA; thus we used multiple enzymes to generate OGAB blocks. Through simulation by varying combinations of the enzymes and lengths of possible OGAB blocks, we determined that if the length is 970 bp (50 blocks), all of the OGAB blocks can be assigned any of the enzymes AarI, BbsI, and BsmBI that do not cut the block (Fig. 3A). Thus we determined that the enzymes AarI, BbsI, and BsmBI were appropriate for use and that the number of OGAB blocks should be 50. The lambda phage DNA was then virtually divided at every 970 bp. The dissection site was named the ideal dissection border. These OGAB blocks were numbered 01, 02, …, 49, and 50, in order from nucleotide numbers 1 to 48526 (Fig. 3A). In the case of seamless assembly, all of the possible protrusion candidates listed above should be searched from the target sequence. However, it would be almost impossible to assign all of the protrusions properly just on the ideal dissection border. Thus we searched for protrusion candidates from a window, which is a sequence around the ideal dissection border with a certain width in bp (Supplemental Table S1). Since the width of the window might affect the size deviation of the OGAB blocks, a short window was preferable. We calculated a smaller window size in the following procedure (Supplemental Table S2). Initially, all of the windows were set to 4 bp and the individual protrusion candidates were counted. If there was a window that had no candidate, each window was expanded 1 bp in both the right and left directions. For example, the ideal dissection border between OGAB blocks 1 and 2 is between absolute nucleotide numbers 970 and 971. In the case of the 12-bp window around the ideal border, i.e., 965-GCTGCT|GGGTGT-976, where | indicates the ideal dissection border, the window contains six protrusion candidates (5’-ACAC-3’ / GTGT-3’, 5’-AGCA-3’ / 5’-TGCT-3’, 5’-CACC-3’ / 5’-GGTG-3’, 5’-CAGC-3’ / 5’-GCTG-3’, 5’-CCAG-3’ / 5’-CTGG, 5’-CTGC-3’ / 5’-GCAG-3’). However, in the case of an ideal dissection border between OGAB blocks 24 and 25, the 12-bp window is 23285-TCTTTT|AATTTT-23286 and there is no protrusion candidate. As a consequence, the first protrusion candidate for this dissection border appeared when the window size was expanded to 24 bp (Supplemental Table S1 and S2). After the window width was determined, we searched for the actual protrusion candidate in the window by starting from a border having fewer

candidates to a greater number of candidates by assigning a rarer protrusion preferentially. For borders between pGETS118-AarI and OGAB block 1, and for those between OGAB block 50 and pGETS118-AarI, 5 5ATTA-3’ / 5 ’TAAT-3’ and 5anAAAA-3’ / 5 ’TTTT-3’ were used, respectively. As a result, all OGAB blocks were found to be between 956 and 988 bp; the mean value ± standard deviation (coefficient of variation) was 970.4 ± 6.3 bp (0.65%), and that became 3691.4 ± 6.5 bp (0.18%) in the state of OGAB block plasmids (Supplemental Table S6). These operations were performed by Microsoft Excel software.

Designing OGAB blocks for an artificial mevalonate operon. To construct an artificial mevalonate operon, the *Saccharomyces Cerevisiae* genes ERG10, ERG13, and HMG1 were converted to novel sequences whose codon usage was optimised for *E. coli* expression by synonymous codon substitution. These sequenced attached ribosome-binding sites at each gene upstream were connected in this order to form an artificial operon (Fig. 4A). This hereby *de novo* designed sequence, 5.9 kb in length, was divided into 55 OGAB blocks. In this case, only AarI was used because there was no recognition site in the objective sequence. The protrusions were assigned as described above. As a result, the size of all OGAB blocks fell to between 98 and 117 bp; the mean value ± standard deviation (coefficient of variation) was 108.2 ± 4.5 bp (4.2%), and this became 2828.2 ± 4.5 bp (0.16%) in the state of OGAB block plasmids (Supplemental Tables S3 and S4).

Preparation of OGAB block plasmids for lambda phage genome reconstruction. The OGAB blocks for lambda phage genome reconstruction were amplified by PCR using the primers listed in Supplemental Table S6. PCR was performed using KOD DNA polymerase (Toyobo) under the following conditions: the reaction mixture consisted of 5 µl of 10×KOD Plus buffer Ver. 2, 3 µl of 25 mM $MgSO_4$, 5 µl of dNTP (2 mM each), and 1 µl of KOD Plus, with water added to bring the volume to 50 µl. After denaturing the template DNA at 94˚C for 2 min, the mixture was subjected to 30 cycles of 98˚C for 20 sec, 55˚C for 30 sec, and 72˚C for 1 min. An A-protrusion at the 3’ end was added to the obtained PCR fragment using A-attachment Mix (Toyobo) according to the instruction manual. The obtained DNA was ligated into pMD19 (simple) (Takara) using Mighty Mix ligation mixture (Takara), and then was used to transform each of the *E. coli* strains TOP10, JM109, and DH5α. The obtained plasmid was extracted and sequenced using specific primers. There are six mutations in authentic lambda phage DNA compared to sequence accession number J02459.1: g.138delG, g.14266_14267insG, g.37589C>T, g.37743C>T, g.43082G>A, and g.45352G>A. We reconstructed the lambda phage genome sequence of authentic DNA, except for one nucleotide: In OGAB block 10, we used the synonymous mutation g.9515G>C, which we had obtained by chance, instead of the wild-type sequence (Fig. 3).

Preparation of OGAB block plasmids for the artificial mevalonate operon construction. The OGAB blocks for the artificial mevalonate operon were prepared by the method of Rossi[21] from synthetic oligonucleotides 80 bp in length, followed by PCR amplification of the extended double-stranded DNA fragment as follows. The extension and subsequent PCR reaction were performed seamlessly using KOD DNA polymerase (Toyobo) under the following conditions: the reaction mixture was 2.5 µl of 10×KOD Plus buffer Ver. 2, 1 µl of 25 mM $MgSO_4$, 2.5 µl of dNTP (2 mM each); 0.5 µl of KOD Plus, 17.25 µl of water, 0.25 µl of 100 pmol/µl amplification primer (5'-TAGCACCTGCACGT-3'), and 1 µl of a mixture of 50 fmol/µl of both of the oligonucleotides listed in Supplemental Table S7. After the synthetic oligonucleotides were denatured at 94˚C for 2 min, the mixture was subjected to 30 cycles of 94˚C for 30 sec, 55˚C for 2 sec, and 74˚C for 6 sec, followed by incubation at 74˚C for 7 min. An A-protrusion at the 3' end was added to the obtained PCR fragment using A-attachment Mix (Toyobo) according to the instruction manual. The obtained DNA was ligated into a pMD19 Simple Vector (Takara) using Mighty Mix ligation mixture (Takara), and then was used to transform *E. coli* strain JM109. The obtained plasmid was extracted and sequenced using specific primers.

Plasmid extraction from *B. subtilis*. The alkaline–SDS method described by Bron[26] was used as follows. Colonies on a plate were picked up by a toothpick and inoculated into 2 mL of antibiotic-containing LB medium. After the culture reached the late-log to stationary phase at 37 ˚C, the plasmid copy number was amplified by the addition of IPTG (Isopropyl-β-D-thiogalactopyranoside) to the culture at a final concentration of 1 mM, followed by cultivation for another 3 h. The bacteria were harvested by centrifugation at 15,000 × g for 30 s. The cell pellet was suspended in 100 µl of solution I (50 mM glucose, 25 mM Tris·HCl (pH8.0), 10 mM EDTA (pH 8.0)) containing 10 mg/mL of lysozyme, and incubated at 37 ˚C for 5 min. This solution was added to 200 µl of solution II (0.2 N NaOH, 1% (w/v) sodium dodecyl sulfate) and then agitated gently until it became transparent. The addition of 150 µl of solution III followed by gentle agitation produced a white precipitate. After centrifugation at 20,000 × g for 5 min, the obtained supernatant was transferred to a new tube and then extracted by 450 µl of mixture of phenol: chloroform: isoamyl alcohol (=25:24:1) and centrifuged at 20,000 × g for 5 min. After 320 µl of supernatant was moved to a new tube, 900 µl of 100% ethanol was added. Vigorous mixing followed by centrifugation at 20,000 g for 10 min gave a DNA pellet at the bottom. The pellet was rinsed with 900 µl of 70% ethanol. After complete removal of the liquid by a micropipette, the DNA was dissolved into 25 µl TE (10 mM Tris·HCl, 1 mM EDTA, pH 7.5). Usually, the TE contains 10 µg/ml of RNaseA to digest remaining RNAs. Eight microliters of this sample was used for the appropriate restriction endonuclease analyses.

Sequence confirmation of assembled constructs. Template plasmid DNA was amplified using an Illustra TempliPhi Large Construction Kit (GE Healthcare) according to the instruction manual. DNA

was sequenced by a 3130xL Genetic analyzer with a BigDye Terminator Cycle Sequencing Kit Ver. 3.1 (Applied Biosystems) using sequence-specific primers.

Plaque formation assay. The assembled plasmid containing the lambda phage genome was digested by lambda terminase (Epicenter) as follows. A 20 µl solution containing 100 ng of the relevant plasmid DNA, 2 µl of 10×ME buffer (supplied with the enzyme), 10 mM of ATP, and 1 µl of lambda terminase (2 unit/µl) was incubated for 30 min at room temperature and then purified by phenol:chloroform:isoamyl alcohol (=25:24:1) treatment and ethanol precipitation. After dissolution in 10 µl of TE, 1 µl was used for lambda DNA packaging, for which Gigapack III Plus Packaging Extract (Agilent Technologies) was used according to the instruction manual.

Quantitative real-time PCR. Relative quantification of DNA was performed using the StepOne Plus Real-Time PCR System (Applied Biosystems) using SYBR Premix Ex TaqII (Tli RNaseH Plus) (Takara) as a reaction mixture. For the measurement of OGAB blocks for lambda phage reconstruction, a serial dilution of authentic lambda phage (Toyobo) was prepared for the standard curve. PCR was performed under the following conditions: 1 cycle at 95˚C for 30 s followed by 40 cycles at 95˚C for 5 s and at 60˚C for 30 s. The primers used are listed in Supplemental Table S6.

Algorithm for ligation simulation. Simulation was performed using VBA in Microsoft Excel 2007. A DNA fragment (F) in the virtual ligation reaction was described using three parameters as $F_i(n_i, L_i, R_i)$, where i is the fragment identification number (practically corresponding to the i-th number of the column on the Excel worksheet), n indicates the number of OGAB blocks in one ligating molecule, and L and R represent protrusion sequences at the left end of the OGAB block and the right end of the OGAB block, respectively. If L = R, these two protrusions are defined to be complementary and can be ligated. Before the ligation simulation is started, all OGAB blocks are oriented in the same direction with OGAB blocks in a final construct. Simulation was performed as follows. For the $F_i(n_i, L_i, R_i)$ fragment, another fragment $F_j(n_j, L_j, R_j)$ that satisfied $i \neq j$ was selected by generating a randomised natural number j ($j \leq m$, where m is the number of DNA molecules) by the RAND() command of VBA and was matched. If these two fragments satisfied $L_i = R_j$, meaning that the left end of the F_i fragment could be ligated to the right end of the F_j fragment, then their parameters were converted to $F_{i(new)}(n_{i(old)}+n_{j(old)}, L_{j(old)}, R_{i(old)})$ and $F_{j(new)}(0, 0, 0)$, respectively. On the other hand, if $R_i = L_j$, meaning that the right end of the F_i fragment could be ligated to the left end of the F_j fragment, then the parameters of the two fragments were converted to $F_{i(new)}(n_{i(old)}+n_{j(old)}, L_{i(old)}, R_{j(old)})$ and $F_{j(new)}(0, 0, 0)$, respectively. In the case where both $L_i \neq R_j$ and $R_i \neq L_j$, were satisfied, no ligation occurred and the parameters remained the same [$F_{i(new)}(n_{i(old)}, L_{i(old)}, R_{i(old)})$ and $F_{j(new)}(n_{j(old)}, L_{j(old)}, R_{j(old)})$]. One ligation cycle, which was defined as ligation for F_i, was started from $i = 1$ to m. After one ligation cycle was

finished, all F_i fragments were sorted in descending order according to their L_i values by using the sort command in the VBA macro to exclude $F_i(0, 0, 0)$, and the total number of fragments that were not $F_i(0, 0, 0)$ was counted. The obtained value was set as a new *m* value for the next ligation cycle. Unless otherwise specified, the ligation simulation was performed until *m* reached $m_{100\%}$, where $m_{100\%}$ represents the number of ligation products in one simulation that exhausted all of the canonical ligation pairs, and was calculated by using information on the initial numbers of OGAB blocks as follows: $m_{100\%}$ = (the total number of initial OGAB blocks) – (the total number of less abundant OGAB blocks that might share same ligation junction) + 1. In the case where ligation efficiency = x%, $m_{x\%}$ was calculated as follows: $m_{x\%}$ = (the total number of initial OGAB blocks) – (the total number of less abundant OGAB blocks that might share the same ligation junction) × x / 100 + 1.

Simulation of ligation. The initial data sets of hypothesied OGAB blocks with defined $CV_{mol}(\%)$ were prepared by Excel 2007 as follows. An Assembly scale number of uniform random numbers between 0 and 1 generated by the RAND() command was normalised as a population with average = 0 and distribution = 1. The initial sets for the number of molecules M_{int} for each fragment were calculated as follows: M_{int} = (the normalised uniform random numbers) × (average number of identical OGAB blocks) × $CV_{mol}(\%)$ / 100 + (average number of identical OGAB blocks). Twenty populations that were independently prepared as described above for each $CV_{mol}(\%)$ and for each assembly scale were subjected to the algorithm until the value of *m* reached that of $m_{100\%}$. The data from 20 simulations were summed. The number of ligation products was counted for each value of *n*. The counted value was multiplied with *n* to calculate the cumulative value for OGAB blocks, and was then piled up to generate a 100% piled-up graph by putting the cumulative value from the greater *n* value nearer to the bottom of the graph.

Supplemental Figures

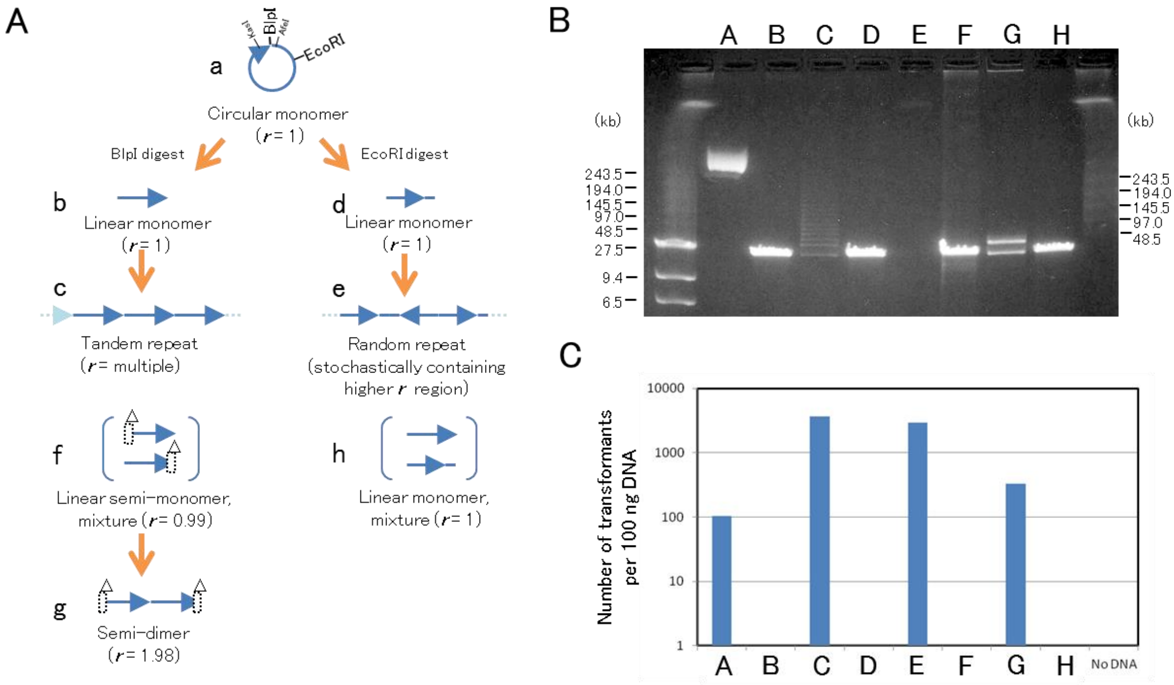

Supplemental Figure S1. Requirement of at least more than one tandem repeat units in a donor DNA in *B. subtilis* plasmid transformation. (A) Structures of plasmid DNAs used in this figure. The plasmid DNA pGETS118-AarI-pBR (a), which appears in Fig. S5, was used as a representative. Restriction digestion of this plasmid with unique enzymes BlpI and EcoRI gave linear monomer plasmid DNAs b and d, respectively, which have *r* (Redundancy of repeat of plasmid DNA unit) = 1. BlpI generates an asymmetric protrusion, and thus the ligation of b forms a tandem repeat product c, which might have a higher *r* value. The ligation product of d, however, does not form tandem repeats but rather random repeat products due to the symmetric protrusion generated by EcoRI. F contained two fragments, KasI-BlpI and BlpI-AfeI, that were dephosphorylated at the KasI or AfeI site, and were used to form G with *r* = 1.98. (B) Pulsed-field gel electrophoresis analysis of substrate DNAs. b, g, f, and h (a mixture of b and d) appear to be equal in size. c, e, and g have ligation products. (C) A number of transformants appeared. No transformant appeared from DNA with *r* = 1, except for a circular monomer. On the other hand, in the case of DNA with *r* > 1, transformants appeared very efficiently.

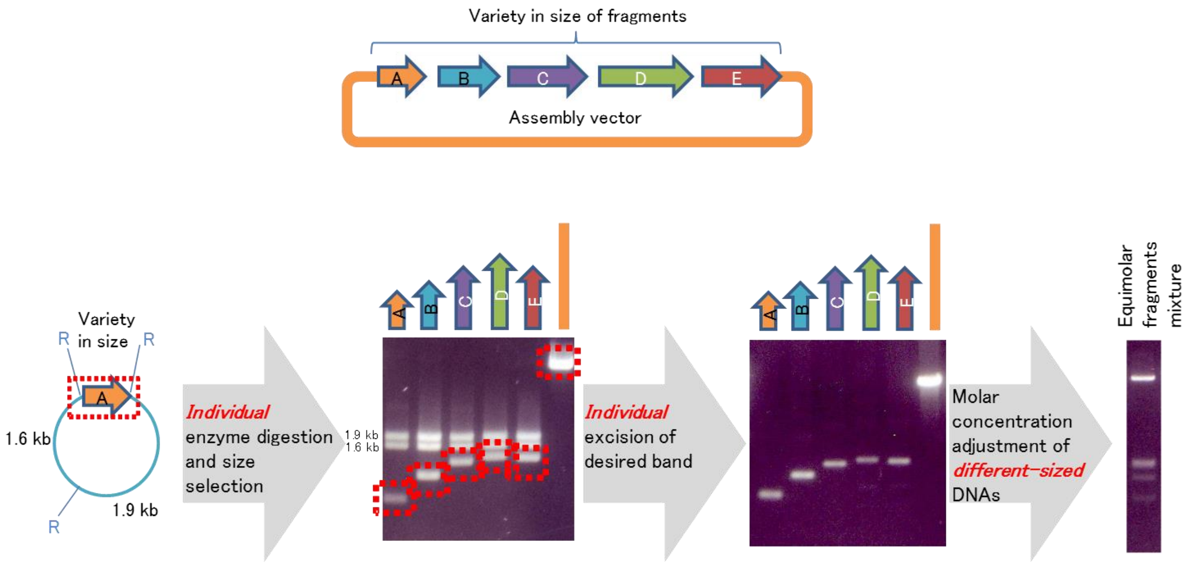

Supplemental Figure S2. Conceptual explanation of the labor-intensive steps in the former OGAB method. The labor-intensive steps required by the original method are shown. A 6-fragment assembly including an assembly vector DNA is shown as an example[5]. In the original OGAB method, the preparation of the DNA material should be performed individually. The workload of these steps is proportional to the number of fragments. Moreover, the molar concentration adjustment of the different-sized fragments would be less accurate.

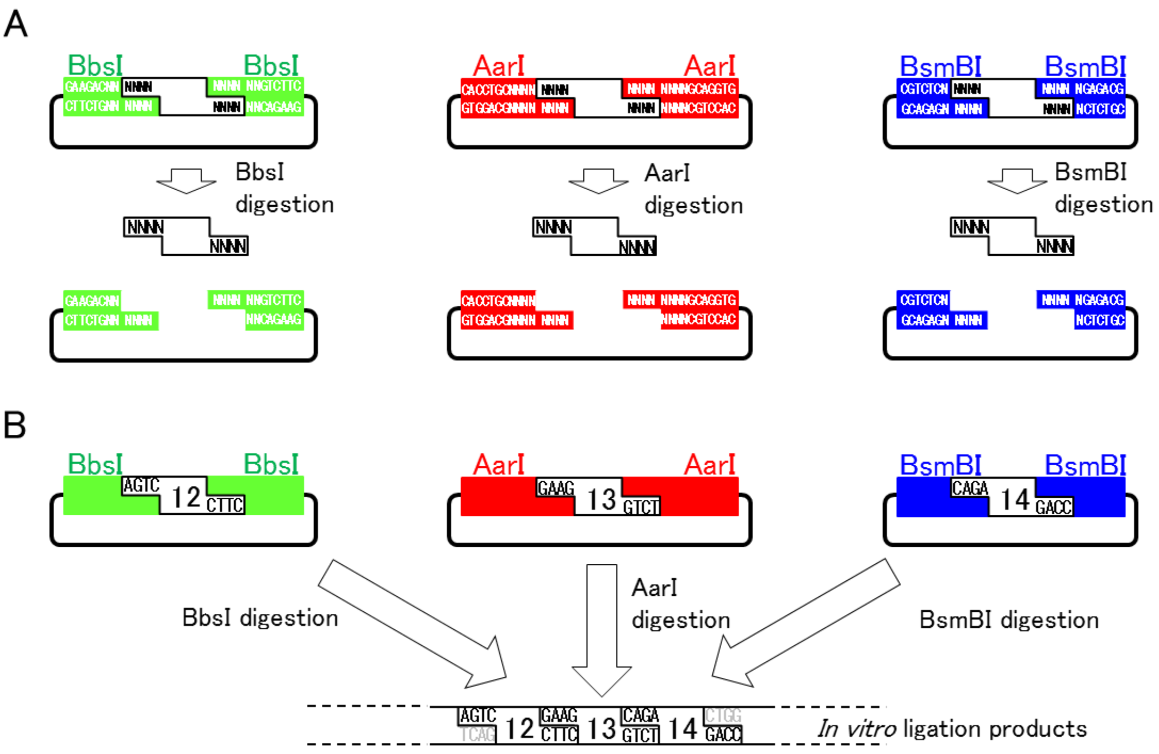

Supplemental Figure S3. Conceptual explanation for the design of protrusions generated by TypeIIS restriction endonucleases. (A) Recognition sequences of TypeIIS restriction enzymes in the OGAB plasmid. BbsI (Left), AarI (Centre), and BsmBI (Right) are indicated by colour. Since these TypeIIS restriction enzymes cut to generate 5'-four nucleotides protruding from either recognition sequence, if two recognition sequences are arrayed in a convergent manner against OGAB blocks, the protrusion sequences of both ends of the relevant blocks can be designed in an arbitrary fashion (denoted as "N" in outlined boxes). (B) Actual examples of protrusion design. Three of the sequential OGAB blocks for lambda phage reconstruction are picked up for explanations. By designing protrusions, we can ligate these OGAB blocks properly without leaving any trace of recognition sequences even though each is generated by a different enzyme.

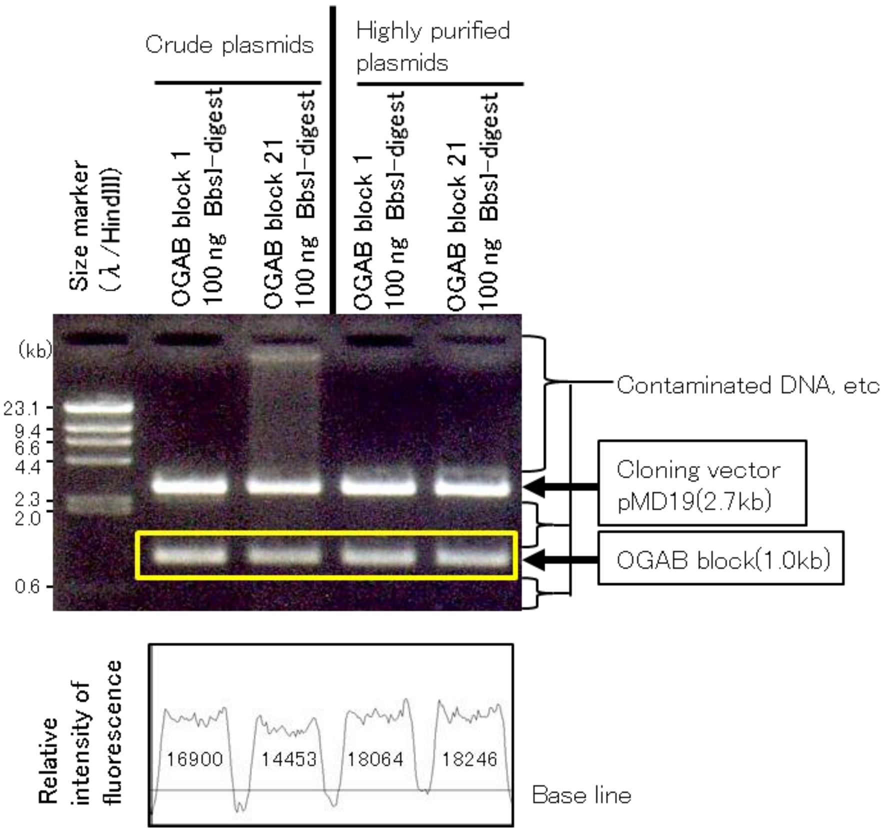

Supplemental Figure S4. The necessity of preparing highly purified plasmid DNA. The purity of subcloned plasmid DNAs before and after Plasmid Safe treatment were compared. Two representative subclone plasmids OGAB block 1 and 21, as average and crude respectively, were selected. The initial total DNA weight concentration of each plasmid solution was measured by a microvolume spectrophotometer NanoDrop-2000. One hundred nanograms of each plasmid before Plasmid Safe treatment (denoted as crude plasmid) and after Plasmid Safe treatment (highly purified plasmid) was digested by BbsI. The resulting samples were electrophoresed and visualised by ethidium bromide. The plasmids were separated into two fragments: a plasmid vector (2.7 kb) and an OGAB block (1.0 kb). But a smear band other than the two bands was observed in a crude sample of OGAB block 21. The DNA concentrations of OGAB blocks in the relevant bands were compared by the fluorescence intensity of each band. The landscape of the fluorescence intensity was calculated from the yellow rectangular area by using NIH Image J software. The numbers in the band represent the relative areas above the base line. The areas of the highly purified bands were similar to each other and numbered more than 18,000, while those of the crude bands were different from each other and fewer in number than the purified bands.

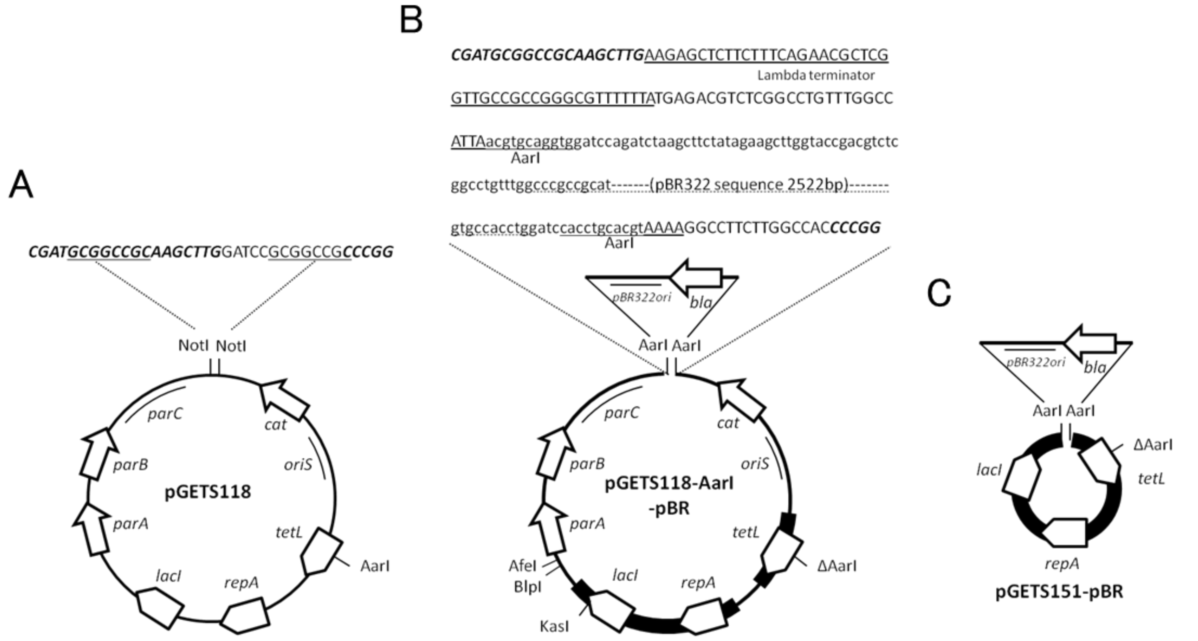

Supplemental Figure S5. Structure of assembly vectors. Plasmid structure used here. (A) Structure of pGET118. This plasmid was constructed from pGETS109 and pBAC108L[26]. The italicised letters indicate a sequence originated from pBAC108L. (B) pGETS118-AarI-pBR was constructed from pGETS118 by several steps. This vector was equipped with an AarI site for gene assembly. The AarI site in the teracycle resistance gene was diminished. (C) Structure of plasmid pGET151-pBR. This plasmid lacked replication ability in *E. coli* cells, and was constructed from pGET118-AarI-pBR by PCR using the OGAB method. Abbreviations: *cat*, chloramphenicol acetyl transferase; *tetL*, tetracycline-resistant gene; *lacI*, repressor gene for *lac* operon of *E. coli*.

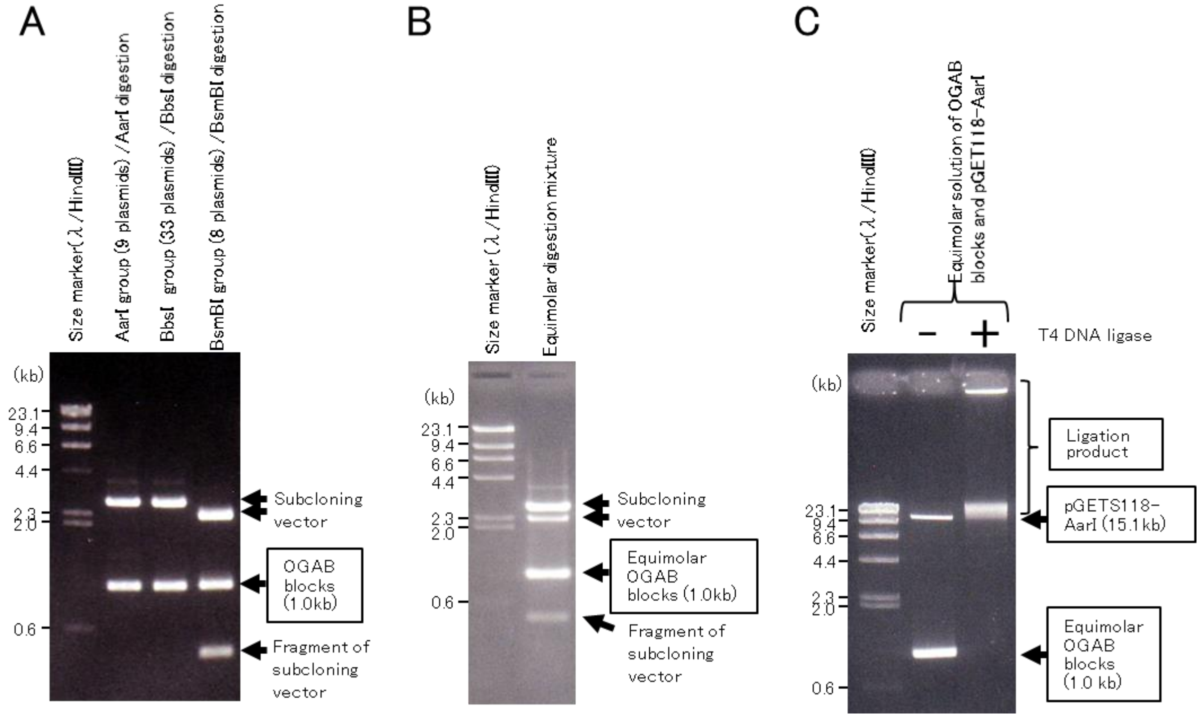

Supplemental Figure S6. Preparation and ligation of OGAB blocks for lambda phage reconstruction. (A) The 50 OGAB blocks for lambda phage were sorted according to the enzyme used. To check the digestion integrity, 5 µl of reaction mixture was electrophoresed. Due to the naturally occurring restriction sites in pMD19, the digest of BsmBI showed two bands other than the OGAB block band. (B) Features of the DNA bands in an actually excised gel. (C) Confirmation of the ligation products. The OGAB block band was no longer in the original position, indicating that the OGAB blocks were ligated almost completely.

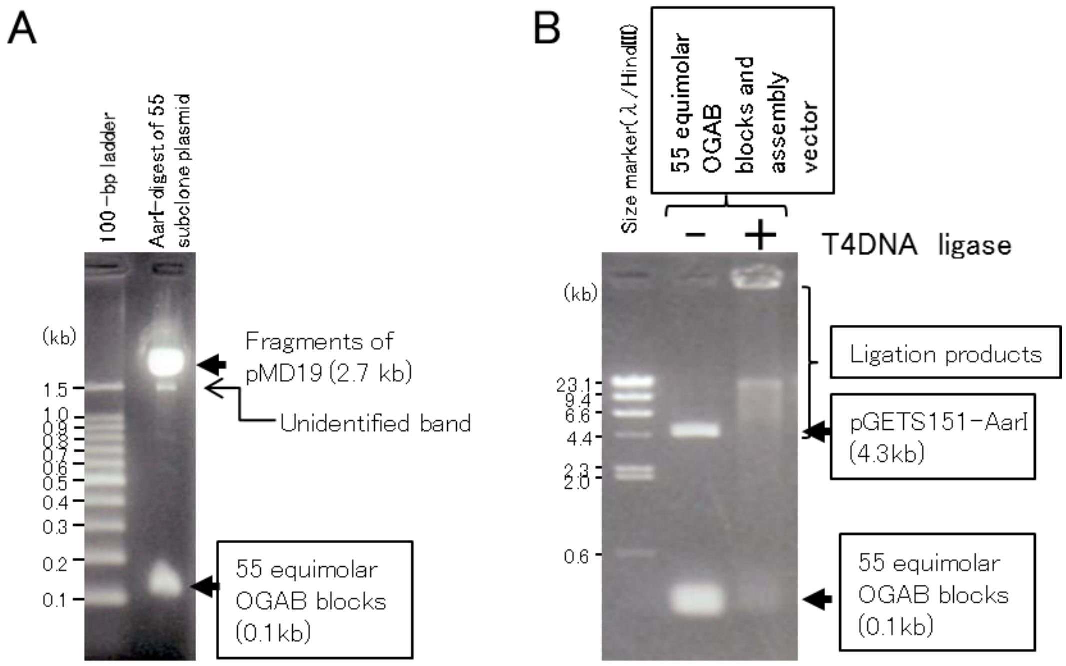

Supplemental Figure S7. Preparation and ligation of OGAB blocks for 55-fragment assembly. (A) Separation of the 55 OGAB blocks in a single electrophoresis. The 0.1 kb band comprised of 55 equimolar OGAB blocks was excised. The thin band below the vector band may have represented an undigested circular subclone plasmid. We ignored this band because the total number of OGAB blocks from possible undigested bands was not particularly large. (B) Electrophoresis analysis of the ligation products. High molecular tandem repeat products appeared in the sample wells, although unligated DNA was observed. These unligated DNAs may indicate that there were a certain number of OGAB blocks that had been denatured at gel extraction.

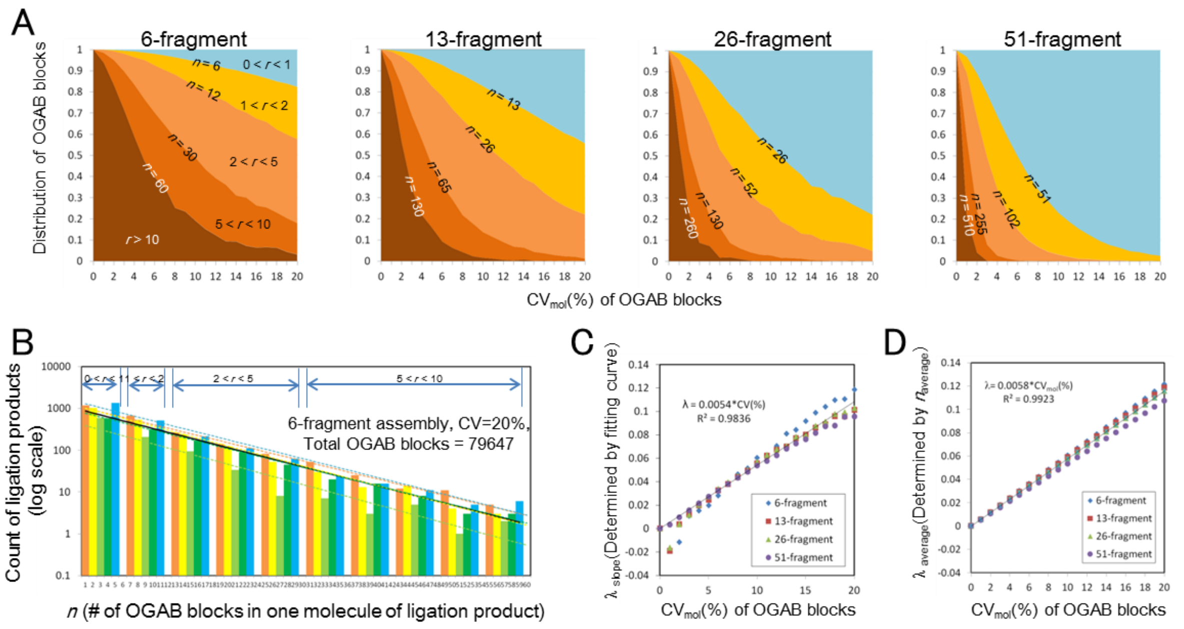

Supplemental Figure S8. Effect of molar deviation of OGAB blocks on the repeat redundancy of the ligation products. (A) Computer simulations of the repeat redundancy distribution of ligation products of OGAB blocks. Four types of assemblies, each incorporating 6, 13, 26, or 51 fragments, were performed. Each simulation was started from an average of 640 hypothetical OGAB blocks with defined CV_{mol} (from 0 to 20%, 1% interval). For each CV_{mol} value of each number-fragment assembly, 20 of the separately prepared randomised initial number sets were used. The simulations were performed under the condition of prohibited circular ligation and were continued until all canonical ligation pairs were exhausted. *r* means the number of repeat redundancies of the ligation product. All the colourings for *r* were the same as those of the 6-fragment assembly. (B) An example of a simulated distribution of ligation products in terms of the number of OGAB blocks in one molecule of ligation product. This figure was constructed using 20 independent simulations under the conditions indicated in the graph area. This distribution profile is quite similar to a geometric or exponential distribution, except for the periodic absence of ligation products where *n* is equal to just multiples of the fragment number of assembly. This phenomenon is reasonable because, under the condition of exhaustive ligation, each ligation product must be surrogated by the surplus OGAB blocks compared with other blocks at both ends, but the ligation product with *n* equal to just multiples of the assembly scale doesn't require this criterion. However, the appearance of zero product peaks prevents the logarithmic transformation of plots that is necessary to calculate the exponential fitting curve. To overcome this issue, we divided each histogram peak according to the remainder of *n* divided by the assembly scale such as the bars having different colours. For each remainder group, data ranging between $0 < r < 10$ (in the case of 6-fragment assembly) were picked and then each rate parameter λ was calculated separately (each fitting curve obtained is denoted as a dashed line coloured the same as the bars). The representative rate parameter λ for each condition was obtained by averaging all the

parameters except for zero remainder bars (black line). (C) Plots of the rate parameter λ_{slope} of exponential distribution that are calculated in the manner explained for the previous figure. All the plots converge to almost the same curve as $\lambda_{slope} = 0.0054 \times CV_{mol}(\%)$, even though there is an 8.5-fold difference in assembly scale. (D) Rate parameter $\lambda_{average}$ determined by $n_{average}$ by Equation 5. The obtained curve ($\lambda_{average} = 0.0058 \times CV_{mol}(\%)$) was almost the same as that determined by the fitting curve ($\lambda_{slope} = 0.0054 \times CV_{mol}(\%)$). Thus we confirmed that the distribution was almost exponential.

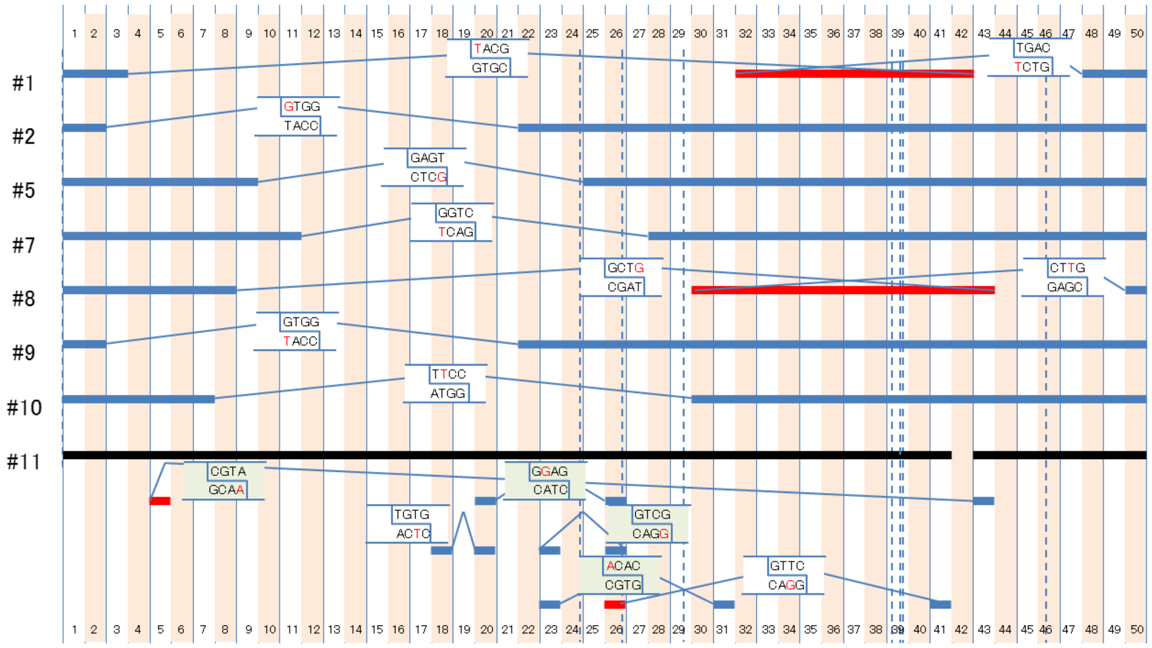

Supplemental Figure S9. Identification of mismatch ligation in wrongly assembled plasmids from lambda genome reconstruction. All of the plasmids from tetracycline-resistance transformants of the lambda phage genome reconstruction were subjected to sequencing to check the precision of the ligation. Except for four constructs that were confirmed to be correctly assembled, all of the clones had more than one misligation. The bold blue lines indicate correctly ligated fragments, while the bold red lines show fragments that were ligated in the opposite direction from the bold blue lines. The bold black lines of #11 indicate structural ambiguity, even though the existence of the OGAB blocks was confirmed. Pairings of protrusions between mismatch OGAB blocks are superimposed between gaps in the OGAB blocks. All of the misligations except for #11 involve T—e.g., T-G or T-T base pairs. Red letters represent mutated nucleotides. In the case of #11, different types of misligation, other than T-related misligations, were observed. These base pairings of protrusions are shaded. Due to the structural ambiguity, #11 was excluded from the calculation of the rate of mismatch ligation.
